# Spontaneous bilateral pneumothorax and pneumomediastinum independent of mechanical ventilation as complications of COVID-19 pneumonia: A case report

**DOI:** 10.5339/qmj.2022.19

**Published:** 2022-06-04

**Authors:** Matthew Antony Manoj, Basavaprabhu Achappa, Nikhil Victor Dsouza, Shruti Sundar, Vinay BS

**Affiliations:** ^1^Department of Internal Medicine, Kasturba Medical College, Mangalore, Manipal Academy of Higher Education, Manipal, India E-mail: basavaprabhu.a@manipal.edu; ^2^Department of Internal Medicine, Kasturba Medical College, Mangalore, Manipal Academy of Higher Education, Manipal, India; ^3^Department of Radiodiagnosis, Kasturba Medical College, Mangalore, Manipal Academy of Higher Education, Manipal, India

**Keywords:** COVID-19, Pandemic, Pneumothorax, Pneumomediastinum, Respiratory complication

## Abstract

Since the beginning of the COVID-19 pandemic, several infected patients have suffered from unusual and severe complications. Among these rare complications, pneumothorax and pneumomediastinum have attracted clinical attention. Such complications might be challenging to diagnose immediately because of the atypical presentation in some cases. Accurate diagnosis is essential to ensure effective treatment. Here, we present the case of a 62-year-old male who presented with symptoms of COVID-19 acute respiratory distress. There was diffuse subcutaneous emphysema in the face, neck, shoulder, and chest wall on clinical examination. The patient was started on oxygenation via a non-rebreather mask. On further evaluation, his chest X-ray revealed bilateral peripheral opacities in the lung fields, bilateral pneumothorax, and subcutaneous emphysema, after which intercostal drainage tubes were inserted. The patient's oxygen saturation was not satisfactory, and he was switched to a high-flow nasal cannula. However, his condition deteriorated, and he was put on mechanical ventilation and inotropic support. Despite our best efforts, the patient succumbed to the disease. We want to emphasize the importance of this adverse event despite his nonsmoking history and the exclusion of positive pressure ventilation. Although benign, early diagnosis of this condition is vital, as the condition could lead to worse morbidity if left unrecognized.

## Introduction

In December 2019, doctors from Wuhan encountered various cases of pneumonia with an unknown etiology. This unknown etiology was identified and named severe acute respiratory syndrome coronavirus 2 (SARS-COV-2), a novel coronavirus, and the disease caused by this virus was named coronavirus disease (COVID-19). Despite the measures taken to minimize the spread of the virus, COVID-19 cases continued to increase worldwide, leading to a global pandemic^1^. As of January 2022, the World Health Organization (WHO) reported 328 million confirmed cases worldwide and 5.5 million deaths. With the rise of the second wave, India has witnessed 34 million cases and 465,000 reported deaths since the beginning of the pandemic^2^.

COVID-19 has been related to a broad spectrum of complications, leading to increased morbidity and mortality. Among these rare complications, pneumothorax and pneumomediastinum have attracted clinical attention. The pathophysiological mechanism of pneumomediastinum can be explained by the development of an increased pressure difference between the marginal alveoli and lung parenchyma, which leads to air in the presence of an external alveolar injury leaking into the interstitium, following the bronchovascular bundle, and spreading through the mediastinum^3^. The exact pathogenesis of pneumothorax occurrence remains unknown. Pneumothorax occurs due to a communication between the pulmonary alveoli and the pleural cavity resulting from air migrating from the alveoli to the pleural cavity until the lung collapses and equilibrium is achieved, or the rupture is sealed^4^. Similarly, when there is communication between the pleural cavity and the thoracic cavity, air moves from the pleural cavity to the thoracic cavity until the pressure difference is equal or until the communication is closed. The main physiological change observed in pneumothorax is the reduction of arterial oxygen and vital lung capacity^4^.

Most clinicians believe that primary spontaneous pneumothorax (PSP) occurs due to spontaneous rupture of a subpleural bleb or bullae. The development of blebs and bullae occurs in an estimated 15% of normal subjects. Its etiology may be attributed to various factors, including genetic predisposition, distal airway inflammation, anatomical bronchial tree abnormalities, apical ischemia at the apices, and abnormal connective tissue, to name a few^5,6^.

Pneumomediastinum can occur due to traumatic injuries, chronic respiratory diseases like chronic obstructive pulmonary disease, bronchiectasis, interstitial lung disease, or have iatrogenic causes^3^. The causes associated with PSP occurrence are subpleural bleb and bullous ruptures. In contrast, secondary spontaneous pneumothorax occurs in individuals with underlying lung pathology, like chronic obstructive pulmonary disease, tuberculosis, malignancy, and interstitial lung diseases, to name a few. Pneumothorax can also occur due to blunt trauma, penetrating trauma, or iatrogenic causes^4-6^.

Some studies have reported the presence of pneumomediastinum independent of barotrauma. This questions whether the presence of specific pathophysiology, mechanism, or injury was involved in developing this complication^7^. Here, we present an unusual case of spontaneous bilateral pneumothorax and pneumomediastinum in COVID-19 pneumonia independent of mechanical ventilation, with no history of lung pathology or smoking, suggesting an alternate pathology.

## Case Presentation

A 62-year-old male presented to the hospital with a history of fever and cough for 5 days associated with 2 days of worsening non-exertional shortness of breath with a medical history of type 2 diabetes mellitus and hypertension. The patient had a history of travel via public transportation 6 days before the admission. The patient denied other COVID-19 symptoms. He does not consume alcohol, smoke tobacco, or use recreational drugs. The physical examination revealed a patient in mild respiratory distress with swelling of the face, neck, and eyelids. Diffuse subcutaneous emphysema was present in the face, neck, shoulder, and chest wall. His oxygen saturation was 84% in room air, which improved to 94% with a non-rebreather mask. His respiratory rate was 28 breaths/min. His temperature was 36.6**°**C (98**°**F), pulse 96 bpm, and blood pressure 168/64 mm Hg. On auscultation, crepitus was present, and the rest of the physical examinations were normal.

**Figure 1.**
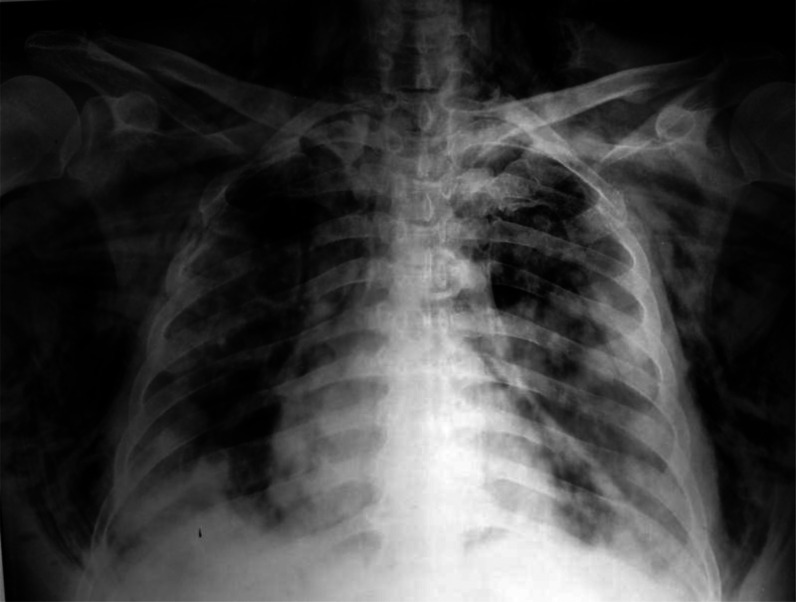
Chest radiograph showing bilateral peripheral opacities in the lung fields, bilateral pneumothorax, and subcutaneous emphysema.

**Figure 2.**
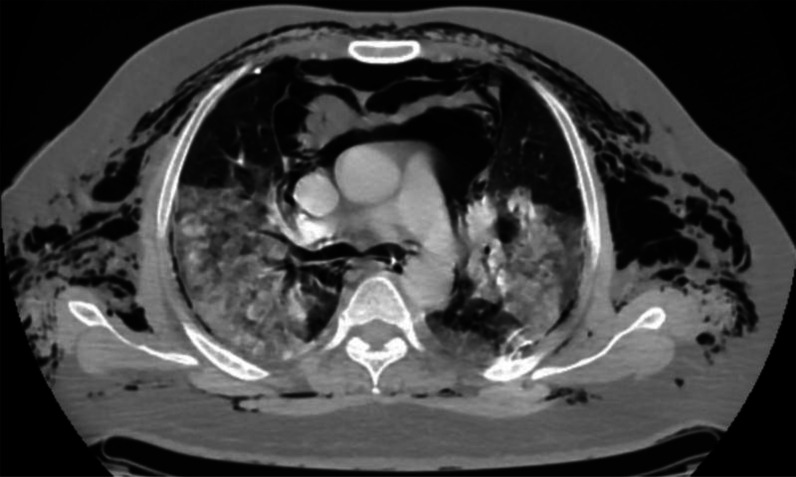
Axial CT image with HRCT window showing extensive subcutaneous emphysema in the chest wall on both sides and extensive air foci seen in the mediastinum.

**Figure 3.**
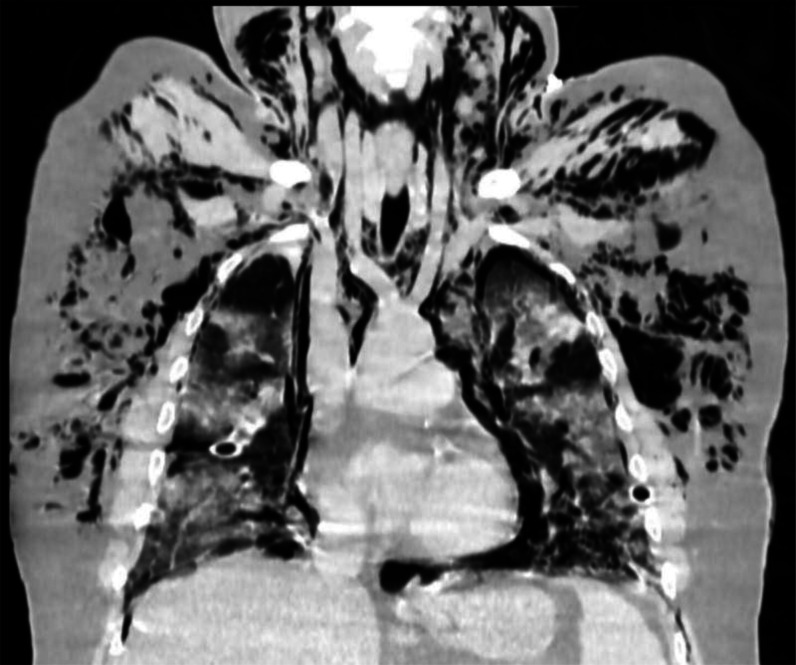
Coronal HRCT window image showing air foci extending above the root of the aorta into the neck region suggestive of pneumomediastinum.

The patient was stabilized and admitted to the intensive care unit (ICU), after which his baseline laboratory tests and radiological investigations were done. His chest X-ray showed bilateral peripheral opacities in the lung fields, bilateral pneumothorax, and subcutaneous emphysema (Figure 1), after which intercostal drainage tubes (ICD) were inserted. A contrast-enhanced computed tomography thorax was obtained, which revealed right-sided minimal hydropneumothorax, left-sided minimal pneumothorax, bilateral peripheral ground-glass opacities with air bronchograms, suggestive of atypical viral pneumonia with computed tomography (CT) severity score of 23/25. Extensive pneumomediastinum was also dissected through the fat planes (Figures 2 and 3). His electrocardiography (ECG) confirmed sinus tachycardia (heart rate = 124 bpm). Reverse transcriptase-PCR (RT-PCR), inflammatory markers, and relevant investigations were performed (Table 1). His arterial blood gas (ABG) profile revealed type 2 respiratory failure, and his RT-PCR was positive for COVID-19.

The patient was transferred to the COVID-19 ICU, and treatment was started with steroids, anticoagulants, remdesivir, and other supportive treatments. Despite the treatment and intercostal drain (ICD) insertion, his oxygen saturation was unsatisfactory (SpO_2_ = 78%), and the patient was switched to a high-flow nasal cannula with the settings titrated as per his ABG. His SpO_2_ was slightly improved (82%). In the following days, his condition worsened (SpO_2_ = 74%, heart rate = 138 bpm, BP = 80/60 mm Hg, and he was drowsy). Hence, the patient was intubated and put on a mechanical ventilator and inotropic support.

Over the following days, the patient's oxygen saturation dropped despite being on ventilatory and inotropic support. Due to acute respiratory distress syndrome and sepsis with septic shock complications, the patient succumbed to the disease.

## Discussion

COVID-19 is a new infectious disease that is a member of the family “Coronaviridae,” which emerged as a global threat and led the WHO to declare it a pandemic in early March 2020. COVID-19 remains a topic of active research as researchers worldwide are actively studying the natural history of the disease^2^. Although individuals affected with SARS-COV-2 have mild symptoms, it can cause severe respiratory symptoms in the elderly population with underlying comorbidities. With the rise of new double-mutant variants of SARS-COV-2, the younger population are now at risk^2^. One such variant is the B.1.617 double-mutant variant, which is responsible for the deadly second wave in India, leading to an incidence of 700,000 cases per week^2^.

COVID-19 has been associated with numerous complications across various body systems. Pneumothorax is a common COVID-19 complication in patients under invasive ventilation. However, only a handful of spontaneous bilateral pneumothorax and pneumomediastinum cases have been reported during the pandemic. The incidence of pneumomediastinum was 1 in 32,896 cases in the general population, whereas the incidence of pneumothorax is 6 in 100,000 population^8,9^. The exact incidence of pneumothorax and pneumomediastinum in COVID-19 is unknown, but specific literature has reported the incidence to be 12%–24% and 13%, respectively^10,11^.

The patient in our case study had no prior risk factors for developing pneumomediastinum and was not treated by positive pressure ventilation before its development. This is in contrast to recent studies by Volpi et al. where the patient developed pneumomediastinum after positive pressure ventilation^12^. A similar study was published by Chu et al. where a COVID-19 positive patient developed pneumomediastinum and subcutaneous emphysema during hospitalization^9^. The patient in our study had presented with spontaneous bilateral pneumothorax and pneumomediastinum during the early stages of the disease.

Dyspnea and chest pain are the most frequent patient complaints. However, at the time of presentation, our patient had mild symptoms resembling “silent hypoxia,” which is atypical of the usual disease manifestations. Chest CT is vital in diagnosing these conditions because of its high sensitivity and negative predictive value^13^. Pneumothorax and pneumomediastinum represent the late sequelae of COVID-19, which, if unnoticed, could lead to fatal consequences. However, in our case, the patient had an early sequela^14^. Although pneumomediastinum is usually benign, it could lead to severe conditions like tension pneumothorax, significant subcutaneous emphysema, and cardiac tamponade, to name a few^13^.

The role of extracorporeal membrane oxygenation (ECMO) for patients with COVID-19 is evolving. Early in the pandemic, the data on ECMO use was limited. An international cohort study done by Barbaro et al. found that patients with COVID-19 treated on ECMO had a mortality rate of 51.9%^15^. However, a recent multicenter study of patients with COVID-19–related ARDS managed with ECMO revealed an estimated 60-day mortality of 31%^16^. Data from the Extracorporeal Life Support Organization registry reported that the 90-day mortality after ECMO initiation is 37.4%^16^. ECMO is indicated in patients with acute respiratory distress syndrome associated with pneumonia, refractory cardiogenic shock, cardiomyopathy, and cardiotoxicity, to name a few. It is contraindicated in patients with chronic organ dysfunction, severe chronic lung disease, and in patients who have been on prolonged mechanical ventilation^16^. The patient in our study was not a candidate for ECMO as he had a bilateral ICD inserted and was on mechanical ventilation. In addition, an ECMO facility was not available at our hospital or in our city. Specialized care was provided to the patient and comprised a team of doctors from the departments of internal medicine, pulmonology, critical care, and infectious diseases.

Conservative treatment is the treatment of choice for pneumomediastinum, and it consists of bed rest with oxygen inhalation, prophylactic antibiotics, and analgesic medications^17^. The patient should be cautioned against coughing and straining, leading to increased intra-thoracic pressure. In addition to this, 100% oxygen therapy has also proven to be effective^14^. We want to emphasize that pneumomediastinum is usually a late sequela or complication of COVID-19 pneumonia. If not identified and treated on time, it can occur, acutely causing management difficulties and mortality.

## Conclusion

Pneumothorax and pneumomediastinum are rare, life-threatening complications of COVID-19. Although pneumomediastinum is reported as a late sequela or complication of COVID-19 pneumonia, this case highlights the importance of identifying pneumomediastinum as an early sequela of COVID-19 pneumonia. If not identified, it can cause difficulties in managing the condition, leading to mortality.

## Figures and Tables

**Table 1:** Patient laboratory investigations

Investigation (units)	Result	Reference range

Hemoglobin (gm/dL)	12.9	12.0–16.0

White cell count (Cells/cumm)	4500	4000–10000

Neutrophils (%)	75.0	40.0–75.0

Lymphocytes (%)	20.2	20.0–40.0

Platelet count (Cells/cumm)	206000	150000–410000

Serum Creatinine (mg/dL)	1.35	0.40–1.40

C reactive protein (mg/L)	20.8	>5 mg/L is positive

Lactate dehydrogenase (IU/L)	252	0.0–250.0

d-dimer (μg/L)	0.60	0.00–0.50

Ferritin (μg/L)	269	13.00–150.00

